# *Moringa oleifera* Supplemented Diets Prevented Nickel-Induced Nephrotoxicity in Wistar Rats

**DOI:** 10.1155/2014/958621

**Published:** 2014-09-11

**Authors:** O. S. Adeyemi, T. C. Elebiyo

**Affiliations:** Department of Biological Sciences, Landmark University, Omu-Aran 370102, Kwara State, Nigeria

## Abstract

*Background*. The *Moringa oleifera* plant has been implicated for several therapeutic potentials. *Objective*. To evaluate whether addition of *M. oleifera* to diet has protective effect against nickel-induced nephrotoxicity in rats. *Methodology*. Male Wistar rats were assigned into six groups of five. The rats were given oral exposure to 20 mg/kg nickel sulphate (NiSO_4_) in normal saline and sustained on either normal diet or diets supplemented with *Moringa oleifera* at different concentrations for 21 days. 24 hours after cessation of treatments, all animals were sacrificed under slight anesthesia. The blood and kidney samples were collected for biochemical and histopathology analyses, respectively. *Results*. NiSO_4_ exposure reduced the kidney-to-body weight ratio in rats and caused significant elevation in the levels of plasma creatinine, urea, and potassium. Also, the plasma level of sodium was decreased by NiSO_4_ exposure. However, addition of *M. oleifera* to diets averted the nickel-induced alteration to the level of creatinine and urea. The histopathology revealed damaged renal tubules and glomerular walls caused by NiSO_4_ exposure. In contrast, the damages were ameliorated by the *M. oleifera* supplemented diets. *Conclusion*. The addition of *M. oleifera* to diet afforded significant protection against nickel-induced nephrotoxicity.

## 1. Introduction

The* Moringa oleifera* is a significant medicinal plant belonging to the family Moringaceae. The* M. oleifera* is recognized for its vast therapeutic properties since ancient times. It is also known as drumstick tree or horseradish tree; the leaves are very beneficial and offer important source of beta-carotene, vitamin C, protein, iron, and potassium [1]. The* M. oleifera* plant is native to the Indian subcontinent and has been used by the Indians for almost 5000 years [2].* M. oleifera* tree can grow well in the humid tropic or hot dry land and it can survive in harsh climatic condition including destitute soil [2, 3]. The root, bark, gum, leaf, pods, flowers, seed, and seed oil are used in traditional medicine for treatment of various ailments [4]. The leaves, flowers, roots, gums, fruits, and seeds of* M. oleifera* are extensively used in the treatment of inflammation, cardiovascular dysfunction, liver disease, and hematological and renal malfunction [5–7]. The leaves of* M. oleifera* (g/g) have the calcium four times that of milk, vitamin C seven times that of oranges, and potassium three times that of bananas, three times the iron of spinach, four times the vitamin A in carrots, and two times the protein in milk [8, 9]. Studies have attributed the medicinal benefits of* M. oleifera* to its anti-inflammatory, antioxidant, and antipathogenic constituents [10, 11]. Also, the strong antioxidant and scavenging ability of* M. oleifera* has been linked to chemoprevention of diseases like cancer [12].

Research efforts have continued to unearth the medicinal and nutritional potential of* M. oleifera* in several significant ways. The* M. oleifera* plant has been used as antispasmodic, stimulant, cough expectorant, and diuretic agent [13]. The use of* M. oleifera* as diuretic agent is applicable in the treatment of prostatitis, kidney stones, bladder ache, scalding urine, water retention, and obesity [14]. In a separate study, the extract of* M. oleifera* showed antinephrotoxic effect against DMBA-induced renal cancer [15]. In a similar work by Owolabi et al., [16],* M. oleifera* extracts reduced the severity of lead-induced nephrotoxicity.

In furtherance of exploring and accentuating the medicinal potential of* M. oleifera* in protein against heavy metal toxicity, the present study evaluated the nephroprotective capacity of* M. oleifera* supplemented diets in Wistar rats orally administered with nickel sulphate (NiSO_4_).

## 2. Methods

### 2.1. Chemicals and Reagents

All chemicals and reagents used were of analytical grade. Commercial reagent kits for the determination of plasma urea, creatinine, potassium, sodium, and protein were either as supplied by Randox diagnostic laboratory, Crumlin, UK, or the Agape laboratory, Switzerland.

### 2.2. Experimental Animals

Thirty male rats of Wistar strain of weight between 190 and 200 g were obtained from the Experimental Animal Farm at the Department of Biochemistry, University of Ilorin, Ilorin, Nigeria. The Wistar rats were housed in plastic animal cages in a well-ventilated experimental room. Rats were allowed to acclimatize for a period of 14 days before the commencement of treatments. During the period of acclimatization, the animals had unlimited access to rat feed pellets and clean water. Handling of animals was in accordance with relevant institutional and ethical guidelines as approved for scientific study.

### 2.3. *Moringa oleifera*

The leaves of* Moringa oleifera* were harvested at the Landmark University Farm, Omu-Aran, Nigeria. The leaves were identified and authenticated by Mr. Bolu Ajayi at the Herbarium Unit, Department of Plant Biology, University of Ilorin, Ilorin, Nigeria. Specimen voucher number is VIH 001/1011.

The* M. oleifera* leaves were air-dried and ground into powder. The powdered material was stored at room temperature in cool dry environment until being required for use.

### 2.4. Feed Formulation

The composition of the feed is as shown in Table 1.

### 2.5. Determination of Proximate Composition

Protein content, crude fat and fibre, moisture content, carbohydrate, and ash content were determined following established protocols as described by the Association of Official Analytical Chemists [17].

### 2.6. Experimental Design

Thirty male Wistar rats of weight between 195 and 200 g were randomly distributed into six experimental groups of five and fed as follows:

 control received NiSO_4_ and fed normal rat chow;
 group 2 received NiSO_4_ and fed with 5%* M. oleifera* supplemented diet;
 group 3 received NiSO_4_ and fed with 10%* M. oleifera* supplemented diet;
 group 4 received NiSO_4_ and fed with 15%* M. oleifera* supplemented diet;
 group 5 received normal saline and fed with 15%* M. oleifera* supplemented diet;
 group 6 received normal saline and fed with normal rat chow.

The rats were exposed to daily administration of 20 mg/kg body weight NiSO_4_ by oral gavage. Treatments lasted for 21 days.

### 2.7. Necroscopy

24 hours after the last treatment, the animals were sacrificed under anesthesia in slight diethyl ether. Blood samples were collected by cardiac puncture into clean EDTA bottles. The blood samples were spun at 5000 g for 10 minutes in a refrigerated centrifuge (Anke TDL-5000B, Shanghai, China) to yield the plasma which was used for the biochemical determinations. The kidneys from each animal were excised into iced physiological solution (pH 7.4), weighed immediately, and fixed in buffered formalin for histopathology examinations.

### 2.8. Biochemical Assays

The biochemical indices were determined in rat plasma using a UV/Vis spectrophotometer (Jenway, Staffordshire, UK) where applicable. The levels of rat plasma total protein (TP), creatinine, and urea were determined using Randox assay kits (Crumlin, UK). The rat plasma electrolytes, sodium, and potassium were determined using reagent assay kits by the Agape laboratory (Switzerland). The intraday and interday coefficient of variation (CV) is less than 5%.

### 2.9. Histopathology Examination

The rat kidney was fixed in 10% buffered neutral formalin (BNF) immediately following excision from animals. Fixed tissues were subsequently processed for histopathology examinations as previously described [18]. Capture and scoring for morphological changes were done by a pathologist blind to the treatments, the Pathology Unit, University of Ilorin Teaching Hospital, Ilorin, Nigeria.

### 2.10. Data analysis

Data were analysed on GraphPad Prism 3 (GraphPad Software Inc., San Diego, CA) using the one-way analysis of variance (ANOVA). Post hoc tests were conducted using the Tukey test. Data are reported as mean value ± standard error of mean (SEM). Mean values at *P* < 0.05 are significant.

## 3. Results

### 3.1. Proximate Composition

**Figure 1:**
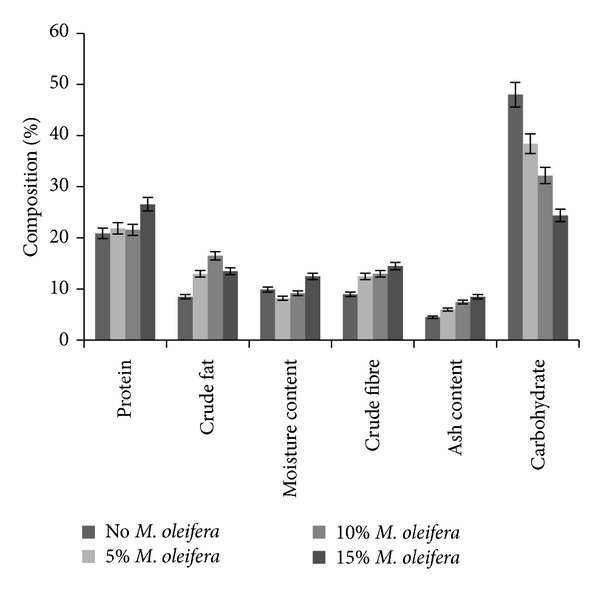
Proximate composition of experimental feeds consisting of various concentrations of* Moringa oleifera* leaves. Data are presented as mean values ± standard error of mean (SEM), *n* = 3.

Diets were formulated consisting of different concentrations of* M. oleifera*. All diets used for the treatment were evaluated for proximate composition by AOAC protocols. The contents of crude fat, fibre, moisture, and ash in the normal rat chow and* M. oleifera* supplemented diets showed no significant difference (Figure 1). However, the carbohydrate content of supplemented formulated diets decreased with increasing concentration of* M. oleifera* relative to the normal diet. Conversely, protein content was higher in the* M. oleifera* supplemented diets.

### 3.2. Kidney and Body Weights

**Figure 2:**
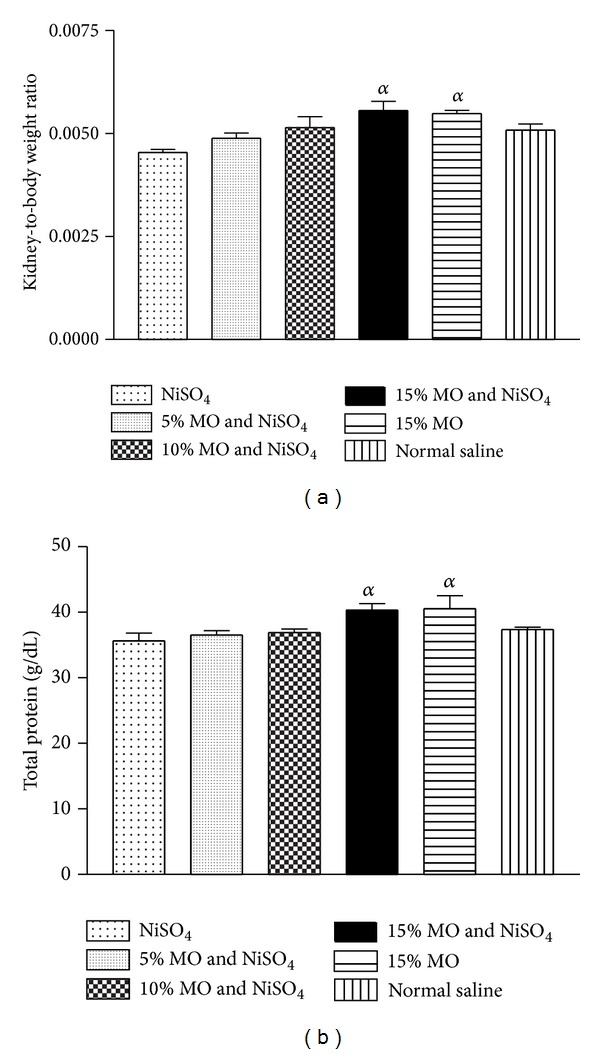
(a) Effect of* Moringa oleifera* supplemented diets on rat kidney-to-body weight ratio following exposure to nickel sulphate; (b) effect of* Moringa oleifera* supplemented diets on levels of rat plasma protein following exposure to nickel sulphate. Data are presented as mean values ± standard error of mean (SEM), *n* = 6.*α* is significantly (*P* < 0.05) relative to nickel sulphate.

The various treatments did not evoke any appreciable change in average rat weights over the course of the exposure (Figure 2). However, there was 12% decline in rat kidney-to-body weight ratio for the group given NiSO_4_ and sustained on normal rat diet. Conversely, there were improvements in the kidney weight with restoration to near normal level for groups maintained on* M. oleifera* supplemented diets.

### 3.3. Plasma Protein

Diets containing the 15%* M. oleifera* caused significant increments in protein level relative to the control group (Figure 2). The other treatment groups did not show any appreciable changes.

### 3.4. Plasma Creatinine

**Figure 3:**
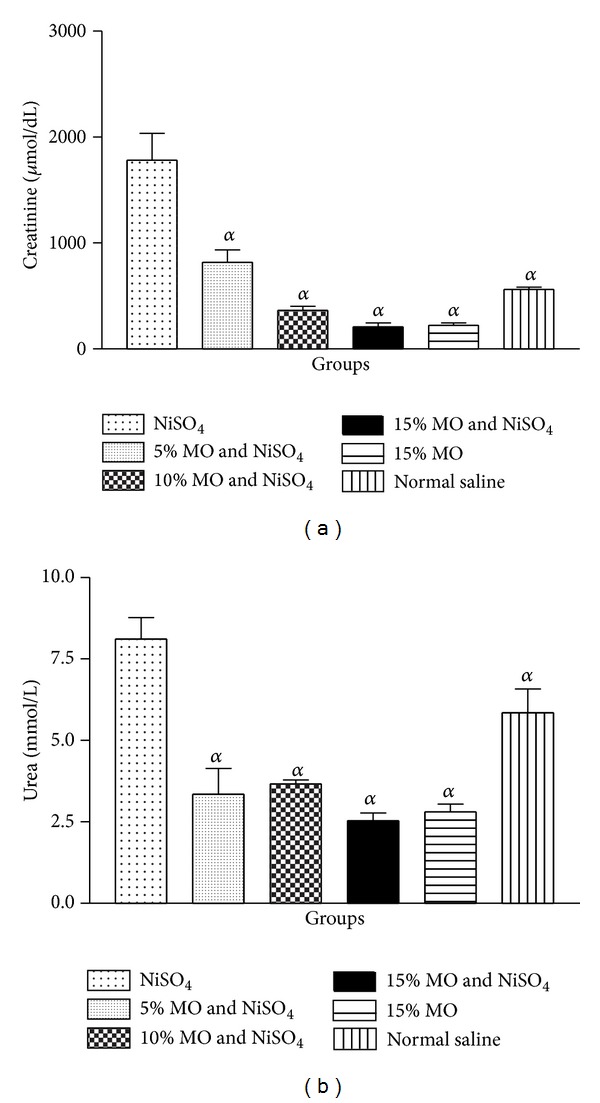
(a) Effect of* Moringa oleifera* supplemented diets on levels of rat plasma creatinine following exposure to nickel sulphate; (b) effect of* Moringa oleifera* supplemented diets on levels of rat plasma urea following exposure to nickel sulphate. Data are presented as mean values ± standard error of mean (SEM), *n* = 6.*α* is significantly (*P* < 0.05) relative to nickel sulphate.

In order to determine whether addition of* M. oleifera* to diets could reverse or modulate the events of nickel-induced nephrotoxicity, the level of plasma creatinine was determined. For the control group which received NiSO_4_ and normal rat diet, the plasma creatinine level was significantly elevated (Figure 3). On the contrary, groups given NiSO_4_ and sustained on* M. oleifera* supplemented diets showed significantly lower plasma creatinine level. The restoration or modulation of the rat plasma creatinine to normal level shows correlation with increasing concentration of* M. oleifera* in the diets given to rats.

### 3.5. Plasma Urea

Furthermore, nickel exposure in the control group sustained on normal diet led to increased (*P* < 0.05) level of plasma urea (Figure 3). On the other hand, rats orally exposed to nickel salt and sustained on* M. oleifera* supplemented diets had significantly reduced level of plasma urea.

### 3.6. Plasma Electrolytes

**Figure 4:**
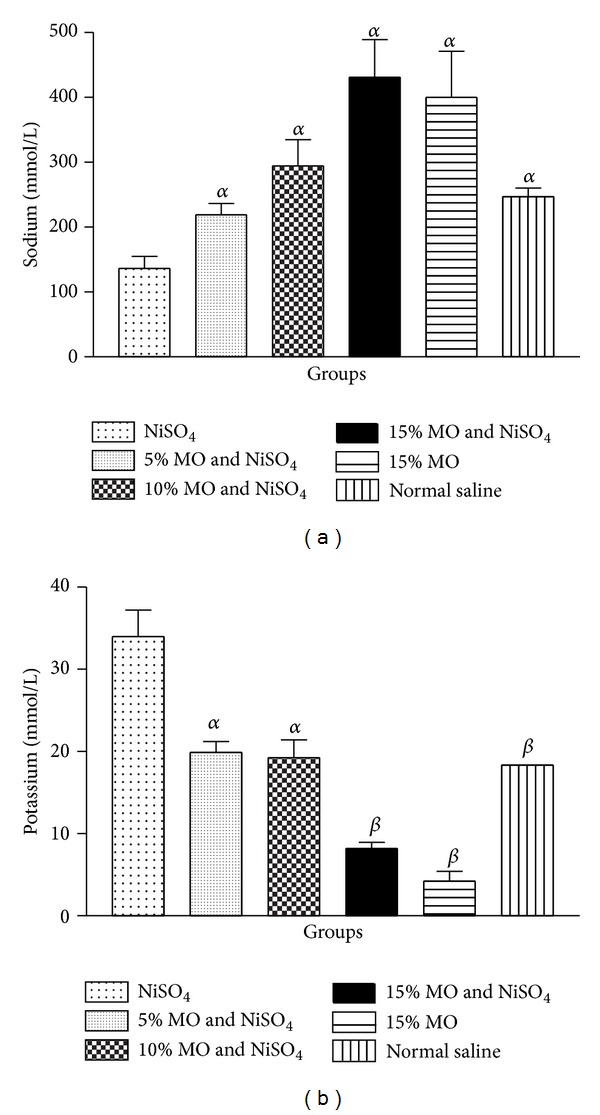
(a) Effect of* Moringa oleifera* supplemented diets on levels of rat plasma sodium following exposure to nickel sulphate; (b) effect of* Moringa oleifera* supplemented diets on levels of rat plasma potassium following exposure to nickel sulphate. Data are presented as mean values ± standard error of mean (SEM), *n* = 6.*α* is significantly (*P* < 0.05) relative to nickel sulphate; *β* is significantly (*P* < 0.01) relative to nickel sulphate.

Oral exposure to NiSO_4_ and daily sustenance on normal diet in the control group for 21 days lowered (*P* < 0.05) the level of rat plasma sodium (Figure 4). However, for rats given NiSO_4_ and sustained on* M. oleifera* supplemented diets, there were significant elevations in levels of plasma sodium.

Also, the control group given NiSO_4_ and normal diet had elevated (*P* < 0.05) level of rat plasma potassium compared to groups sustained on* M. oleifera* supplemented diets in which the rat plasma potassium was appreciably maintained at near normal level.

### 3.7. Histopathology Examinations

**Figure 5:**
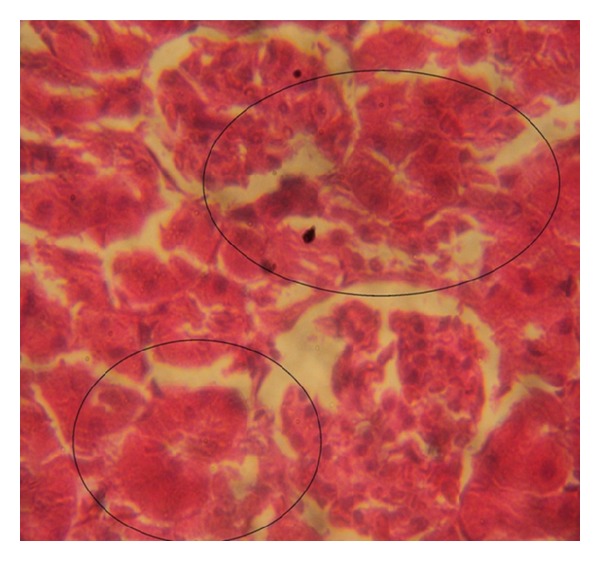
Photomicrographs of rat renal sections following oral exposure to nickel sulphate and sustenance on normal diets. H&E staining (×400).

**Figure 6:**
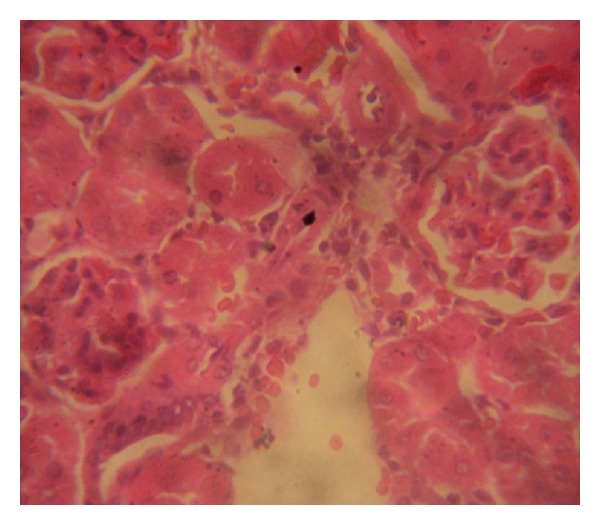
Photomicrographs of rat renal sections following oral exposure to nickel sulphate and sustenance on 5%* Moringa oleifera* supplemented diets. H&E staining (×400).

**Figure 7:**
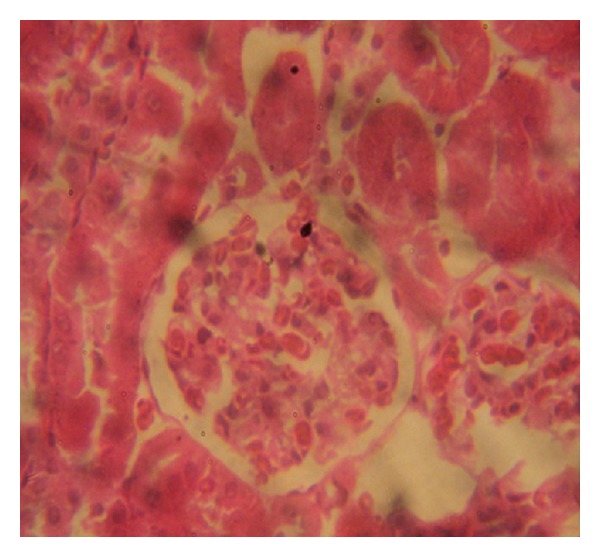
Photomicrographs of rat renal sections following oral exposure to nickel sulphate and sustenance on 10%* Moringa oleifera* diets. H&E staining (×400).

**Figure 8:**
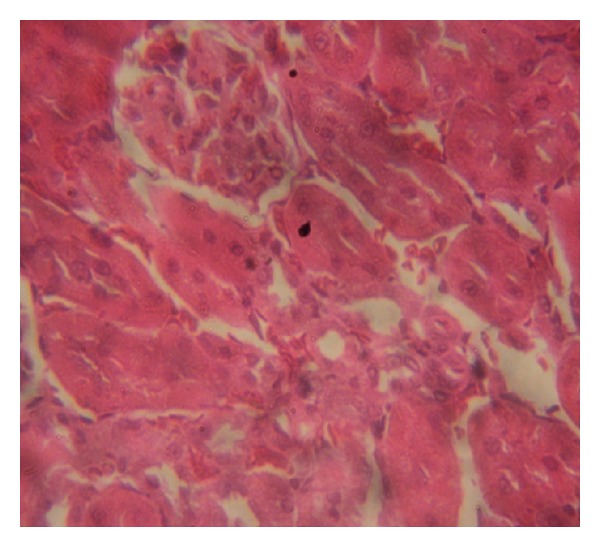
Photomicrographs of rat renal sections following oral exposure to nickel sulphate and sustenance on 15%* Moringa oleifera* diets. H&E staining (×400).

**Figure 9:**
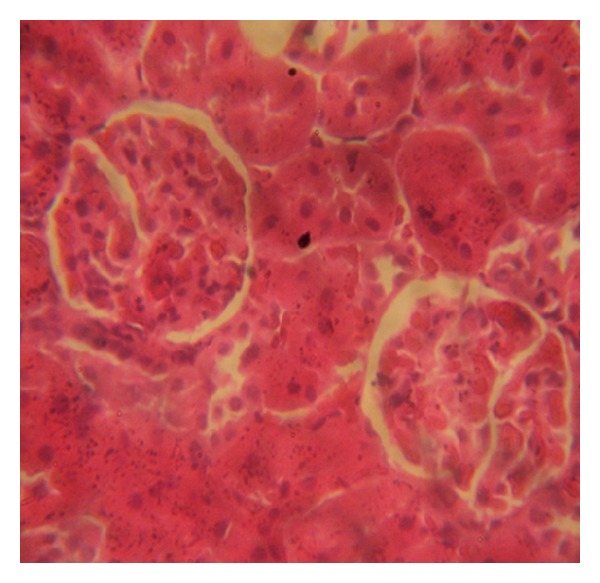
Photomicrographs of rat renal sections following oral exposure to normal saline and sustenance on 15%* Moringa oleifera* diets. H&E staining (×400).

**Figure 10:**
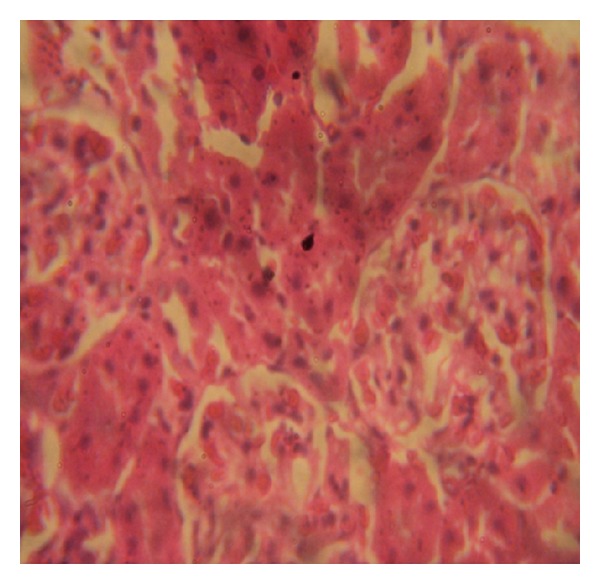
Photomicrographs of rat renal sections following oral exposure to normal saline and sustenance on normal diets. H&E staining (×400).

The examination of rat renal sections for morphological changes revealed inimical cellular alterations in control group caused by NiSO_4_ exposure. The cellular alterations caused included swollen renal tubules, hyaline change, thickening of glomerular wall, mild nephritis, and necrosis (Figures 5, 6, 7, 8, 9, and 10). However, all of the cellular damages caused by NiSO_4_ exposure were nonexistent or ameliorated in the groups sustained on the* M. oleifera* diets.

## 4. Discussion

The* M. oleifera* has shown remarkable potential which could be harnessed for medicinal and nutritional purposes. In recent times, several studies have been able to demonstrate the medicinal significance of* M. oleifera* [19–22]. In separate studies, addition of* M. oleifera* to diet has revealed considerable promises as an adjunct to improving health in a variety of important ways [22, 23].

In furtherance of the evidence of medicinal and nutritional benefits of* M. oleifera*, diets consisting of different concentrations of* M. oleifera* were compounded and evaluated for potential of attenuating nickel-induced nephrotoxicity in rats.

Proximate analysis of the diets revealed higher protein content in the* M. oleifera* supplemented diets. The higher protein content may be due to addition of* M. oleifera* to the diets. Earlier studies have revealed* M. oleifera* as being rich in protein content [8, 9]. To further underscore the nutritional value of* M. oleifera* diets, the proximate analysis showed reduced carbohydrate content with increasing percentage of* M. oleifera*. This may imply nutritional relevance for management of diabetics or related metabolic disorder.

Measurement of body weight and/or organ weight may be used to evaluate toxic events due to exposure to a toxicant. Alteration in body weight and/or organ to body weight ratio has been shown to imply toxicity arising from exposure to a toxicant [24, 25]. No appreciable weight change was recorded for all treatment groups. However, declining kidney-to-body weight ratio in the control group given NiSO_4_ and normal diet was improved significantly in groups exposed to NiSO_4_ and* M. oleifera* supplemented diets. This could be an indication that* M. oleifera* addition to diet was able to attenuate the adverse effect of NiSO_4_ administration on rat renal tissues.

On the other hand, rat plasma protein levels were not appreciably affected by NiSO_4_ exposure. However, sustaining animals on diets containing 15%* M. oleifera* increased the protein level. This may be due to the fact that* M. oleifera* has a high content of protein [8, 9]. This is even more plausible if consideration is given to the proximate composition data in which the 15%* M. oleifera* diet had the highest protein content.

Plasma levels of creatinine and urea are among the major biochemical indices commonly used to evaluate renal functions [18, 26]. Creatinine is a by-product of muscle metabolism and under normal physiological condition the amount excreted per day is constant and correlates with body mass [26]. Conversely, when creatinine level is elevated due to retention in the blood, it could be used to evaluate glomerular filtration rate [18]. Urea, on the other hand, is formed as means to rid body of nitrogenous waste from protein degradation. It is formed in the liver and excreted by the kidney in urine. Elevated plasma urea level has been linked to reduced renal function [18, 26].

In the present study, levels of plasma creatinine and urea were determined. The oral exposure of NiSO_4_ in rats caused significant elevation to the plasma levels of creatinine and urea in a manner reminiscent of compromised renal integrity. However, the feeding of diets containing different concentrations of* M. oleifera* to rats prevented the elevation of the indices for renal dysfunction. These findings are in support of previous studies which showed that* M. oleifera* offered nephro- and hepatoprotection [15, 20, 21, 27]. Also, in a recent study, the protective effect of* M. oleifera* against lead-induced nephrotoxicity has been demonstrated [16]. The evidences are demonstrations which strengthen further the belief that addition of* M. oleifera* to diets could improve health status.

To further evaluate nephroprotective effect of diets containing* M. oleifera*, we determined the level of rat plasma electrolytes. Our results revealed significant alteration to levels of rat plasma electrolytes caused by oral exposure to NiSO_4_. On the contrary, the significant reductions in levels of plasma sodium and elevations in potassium level caused by NiSO_4_ were averted by* M. oleifera* supplemented diets. The maintenance of normal levels of plasma electrolytes is crucial to homeostatic balance. Perturbation of plasma electrolyte balance may affect the pH, osmolality, and blood volume with adverse impact on the kidney and other related body organs.* M. oleifera* has been shown to possess diuretic effect [13, 14] and this may have contributed to protecting against homeostatic imbalance imposed by nickel exposure. Moreover, the maintenance of normal level of plasma sodium and potassium by* M. oleifera* diet is consistent with reported therapeutic potential including the improvement of the health of renal tissues and general well-being [20, 21]. Although, at 10 and 15%* M. oleifera* supplementation, there were increases or decreases in the level of sodium and potassium, respectively, the alterations were found not to be significant when compared to the untreated group given normal saline and 0%* M. oleifera* diet.

The histopathology of renal sections revealed inimical cellular lesions caused by NiSO_4_ exposure in rats. The hyaline change, thickening of glomerular wall, mild nephritis, and necrotic areas caused by NiSO_4_ were conspicuously absent or attenuated in the groups fed on diets containing* M. oleifera*. The histopathology presentations support the biochemical findings and confirm further the medicinal protection afforded by* M. oleifera* against drug-induced tissue damage [15, 16, 19, 21, 27].

To our knowledge, this is the first study which revealed that the addition of* M. oleifera* to diet protected rat kidney against the toxic events caused by NiSO_4_ exposure. The data are further scientific demonstration which underpins the medicinal and nutritional potential of* M. oleifera*. For people working in the mining industries or other related sectors, where the frequency of exposure to heavy metals is high, consumption of diet containing* M. oleifera* may help protect against occupational health risks.

## Highlights

1. Male Wistar rats were daily exposed to oral administration of NiSO_4_ and sustained on either normal diets or* Moringa oleifera* supplemented diets.
2. In the control group which was sustained on normal diets, NiSO_4_ exposure altered the levels of plasma metabolites including urea, creatinine, and electrolytes in a manner that is reminiscent of impaired renal function.
3. In the other treatment groups sustained on* Moringa oleifera* supplemented diets, there were no alterations to the levels of plasma urea, creatinine, and electrolytes revealing nephroprotective potential of the supplemented diets.
4. Oral exposure of NiSO_4_ caused damage to renal tubules and glomerular walls. However,* Moringa oleifera* supplemented diets protected against the renal damage.
5. The addition of* Moringa oleifera* to diet protected against nickel-induced nephrotoxicity.

## Figures and Tables

**Table 1:** Formulation of experimental feed.

Feeds component (g)	Normal feed	5% *Moringa oleifera *	10% *Moringa oleifera *	15% *Moringa oleifera *
Sucrose	300	300	300	300
Cellulose	120	120	120	120
Soybean	750	750	750	750
*Moringa oleifera *	—	300	600	900
Vitamin mix	150	150	150	150
D-methionine	12	12	12	12
Corn starch	1518	1218	918	618
Soybean oil	150	150	150	150

Total	3000	3000	3000	3000

## References

[1] Verma AR, Vijayakumar M, Mathela CS, Rao CV (2009). *In vitro* and *in vivo* antioxidant properties of different fractions of *Moringa oleifera* leaves. *Food and Chemical Toxicology*.

[2] Morton JF (1991). The horseradish tree, *Moringa pterygosperma* (Moringaceae)—a boon to arid lands?. *Economic Botany*.

[3] Anwar F, Ashraf M, Bhanger MI (2005). Interprovenance variation in the composition of *Moringa oleifera* oilseeds from Pakistan. *Journal of the American Oil Chemists' Society*.

[4] Odebiyi A, Sofowora EA (1999). Pytochemical screenings of Nigerian medicinal plants part 11. *Lyodia*.

[5] Mazu̧mder UK, Gupta M, Chakrabarti S, Pal D (1999). Evaluation of hematological and hepatorenal functions of methanolic extract of *Moringa oleifera* Lam. root treated mice. *Indian Journal of Experimental Biology*.

[6] Rao KS, Mishra SH (1998). Anti-inflammatory and antihepatotoxic activities of the rats of moringa pterygosperma geaertn. *Indian Journal of Pharmaceutical Sciences*.

[7] Mahajan SG, Mehta AA (2008). Effect of *Moringa oleifera* Lam. seed extract on ovalbumin-induced airway inflammation in guinea pigs. *Inhalation Toxicology*.

[8] Asante WJ, Nasare IL, Tom-Dery D, Ochire-Boadu K, Kentil KB (2014). Nutrient composition of Moringa oleifera leaves from two agro ecological zones in Ghana. *African Journal of Plant Science*.

[9] Bamishaiye EI, Olayemi FF, Awagu EF, Bamshaiye OM (2011). Proximate and phytochemical composition of Moringa oleifera leaves at three stages of maturation. *Advance Journal of Food Science and Technology*.

[10] Kumar NA, Pari L (2003). Antioxidant action of *Moringa oleifera* Lam. (drumstick) against antitubercular drugs induced lipid peroxidation in rats. *Journal of Medicinal Food*.

[11] Bharali R, Tabassum J, Azad MRH (2003). Chemomodulatory effect of *Moringa oleifera*, Lam, on hepatic carcinogen metabolising enzymes, antioxidant parameters and skin papillomagenesis in mice. *Asian Pacific Journal of Cancer Prevention*.

[12] Atawodi SE, Atawodi JC, Idakwo GA (2010). Evaluation of the polyphenol content and antioxidant properties of methanol extracts of the leaves, stem, and root barks of *Moringa oleifera* Lam. *Journal of Medicinal Food*.

[13] Mbikay M (2012). Therapeutic potential of *Moringa oleifera* leaves in chronic hyperglycemia and dyslipidemia: a review. *Frontiers in Pharmacology*.

[14] Kumar PS, Mishra D, Ghosh G, Panda GS (2010). Medicinal uses and pharmacological properties of *Moringa oleifera*. *International Journal of Phytomedicine*.

[15] Sharma V, Paliwal R (2012). Chemo protective role of *Moringa oleifera* and its isolated saponin against DMBA induced tissue damage in male mice: a histopathological analysis. *International Journal of Drug Development & Research*.

[16] Owolabi JO, Ghazal OK, Williams FE, Gurusa OO Assessment of the prophylactic and rejuvenative effects of moringa oleifera phytochemicals extracts on lead-induced renal tissue disruption in adults male wistar rats models.

[17] AOAC (1995). *Official Methods of Analysis*.

[18] Adeyemi OS, Akanji MA (2012). Psidium guajava leaf extract: effects on rat serum homeostasis and tissue morphology. *Comparative Clinical Pathology*.

[19] Pari L, Kumar NA (2002). Hepatoprotective activity of *Moringa oleifera* on antitubercular drug-induced liver damage in rats. *Journal of Medicinal Food*.

[20] Anwar F, Latif S, Ashraf M, Gilani AH (2007). *Moringa oleifera*: a food plant with multiple medicinal uses. *Phytotherapy Research*.

[21] Ndong M, Uehara M, Katsumata S-I, Suzuki K (2007). Effects of oral administration of *Moringa oleifera* Lam on glucose tolerance in Goto-Kakizaki and wistar rats. *Journal of Clinical Biochemistry and Nutrition*.

[22] Promkum C, Kupradinun P, Tuntipopipat S, Butryee C (2010). Nutritive evaluation and effect of *Moringa oleifera* pod on clastogenic potential in the mouse. *Asian Pacific Journal of Cancer Prevention*.

[23] Johnson BC (2005). *Clinical Perspectives on the Health Effects of Moringa oleifera : A Promising Adjunct for Balanced Nutrition and Better Health*.

[24] Orisakwe OE, Husaini DC, Afonne OJ (2004). Testicular effects of sub-chronic administration of *Hibiscus sabdariffa* calyx aqueous extract in rats. *Reproductive Toxicology*.

[25] Adeyemi OS, Fambegbe M, Daniyan OR, Nwajei I (2012). Yoyo Bitters, a polyherbal formulation influenced some biochemical parameters in Wistar rats. *Journal of Basic and Clinical Physiology and Pharmacology*.

[26] Gross JL, de Azevedo MJ, Silveiro SP, Canani LH, Caramori ML, Zelmanovitz T (2005). Diabetic nephropathy: diagnosis, prevention, and treatment. *Diabetes Care*.

[27] Awodele O, Oreagba IA, Odoma S, da Silva JA, Osunkalu VO (2012). Toxicological evaluation of the aqueous leaf extract of *Moringa oleifera* Lam. (Moringaceae). *Journal of Ethnopharmacology*.

